# Mapping of novel salt tolerance QTL in an Excalibur × Kukri doubled haploid wheat population

**DOI:** 10.1007/s00122-018-3146-y

**Published:** 2018-07-30

**Authors:** Muhammad A. Asif, Rhiannon K. Schilling, Joanne Tilbrook, Chris Brien, Kate Dowling, Huwaida Rabie, Laura Short, Christine Trittermann, Alexandre Garcia, Edward G. Barrett-Lennard, Bettina Berger, Diane E. Mather, Matthew Gilliham, Delphine Fleury, Mark Tester, Stuart J. Roy, Allison S. Pearson

**Affiliations:** 1 Australian Centre for Plant Functional Genomics, PMB 1, Glen Osmond, SA 5064 Australia; 2 School of Agriculture, Food and Wine, The University of Adelaide, PMB 1, Glen Osmond, SA 5064 Australia; 3 Plant Industries Development, Department of Primary Industry and Resources, PO Box 3000, Darwin, NT 0801 Australia; 4 The Plant Accelerator, Australian Plant Phenomics Facility, The University of Adelaide, Urrbrae, SA 5064 Australia; 5 Phenomics and Bioinformatics Research Center, The University of South Australia, GPO Box 2471, Mawson Lakes, 5001 SA Australia; 6 Bethlehem University, Rue de Freres #9, Bethlehem, West Bank Palestine; 7 School of Agriculture and Environment (M084), The University of Western Australia, 35 Stirling Highway, Crawley, WA 6009 Australia; 8 Department of Primary Industries and Regional Development, 3 Baron-Hay Court, South Perth, 6151 WA Australia; 9 ARC Centre of Excellence in Plant Energy Biology, The University of Adelaide, PMB 1, Glen Osmond, SA 5064 Australia; 10 Division of Biological and Environmental Sciences and Engineering, King Abdullah University of Science and Technology, Thuwal, Saudi Arabia

## Abstract

**Key message:**

Novel QTL for salinity tolerance traits have been detected using non-destructive and destructive phenotyping in bread wheat and were shown to be linked to improvements in yield in saline fields.

**Abstract:**

Soil salinity is a major limitation to cereal production. Breeding new salt-tolerant cultivars has the potential to improve cereal crop yields. In this study, a doubled haploid bread wheat mapping population, derived from the bi-parental cross of Excalibur × Kukri, was grown in a glasshouse under control and salinity treatments and evaluated using high-throughput non-destructive imaging technology. Quantitative trait locus (QTL) analysis of this population detected multiple QTL under salt and control treatments. Of these, six QTL were detected in the salt treatment including one for maintenance of shoot growth under salinity (*QG*_*(1*–*5)*_*.asl*-*7A*), one for leaf Na^+^ exclusion (*QNa.asl*-*7A*) and four for leaf K^+^ accumulation (*QK.asl*-*2B.1*, *QK.asl*-*2B.2*, *QK.asl*-*5A* and *QK:Na.asl*-*6A*). The beneficial allele for *QG*_*(1*–*5)*_*.asl*-*7A* (the maintenance of shoot growth under salinity) was present in six out of 44 mainly Australian bread and durum wheat cultivars. The effect of each QTL allele on grain yield was tested in a range of salinity concentrations at three field sites across 2 years. In six out of nine field trials with different levels of salinity stress, lines with alleles for Na^+^ exclusion and/or K^+^ maintenance at three QTL (*QNa.asl*-*7A*, *QK.asl*-*2B.2* and *QK:Na.asl*-*6A*) excluded more Na^+^ or accumulated more K^+^ compared to lines without these alleles. Importantly, the *QK.asl*-*2B.2* allele for higher K^+^ accumulation was found to be associated with higher grain yield at all field sites. Several alleles at other QTL were associated with higher grain yields at selected field sites.

**Electronic supplementary material:**

The online version of this article (10.1007/s00122-018-3146-y) contains supplementary material, which is available to authorized users.

## Introduction

Soil salinity is a significant abiotic stress that limits the production of cereal crops worldwide (Flowers et al. 1997; Munns and Gilliham 2015; Rengasamy 2006; Roy et al. 2014). It has been estimated that more than 20% of irrigated and 8% of rainfed agricultural land is affected by salinity (FAO 2017; Hasegawa 2013; Rengasamy 2006). This area is expected to increase due to changing global climatic conditions, land clearing and irrigation practices causing rising water tables. The deleterious effects of soil salinity on the growth and development of cereals include poor germination rates, reduced plant growth, premature leaf senescence, less tillering and lower grain yields (Fricke and Peters 2002; Munns and Tester 2008; Roy et al. 2014).

To help mitigate the effects of salinity on crop yields, it is important to recognize that a number of sub-traits underpin plant salinity tolerance. To develop high-yielding salt-tolerant cultivars for salt-affected land, crops should contain favourable alleles for a number of these sub-traits (Flowers 2004; Gilliham et al. 2017; Roy et al. 2014). While multiple genes and quantitative trait loci (QTL) have been found to affect plant salt tolerance, there are only a few cases where the results of laboratory or glasshouse studies have been validated under field conditions (Genc et al. 2013; Munns et al. 2012). For results obtained under controlled conditions to be translated into outcomes for growers, it is important to investigate whether specific loci contribute to grain yield improvement under saline field conditions (Gilliham et al. 2017).

Greater salinity tolerance of plants has been reported to be associated with exclusion of Na^+^ and Cl ¯ from the shoot (Genc et al. 2010; Poustini and Siosemardeh 2004; Rashid et al. 1999; Roy et al. 2014; Teakle and Tyerman 2010), maintenance of K^+^ homoeostasis (Chen et al. 2005; Munns and Tester 2008), accumulation of toxic ions in the vacuole (Munns et al. 2016; Munns and Tester 2008), accumulation of compatible solutes (Flowers and Colmer 2008; Hasegawa et al. 2000; Munns and Tester 2008) and/or ability to maintain shoot growth during the early phase of salt stress before ions accumulate to toxic concentrations in the shoot (shoot ion-independent tolerance or osmotic tolerance) (Munns and Tester 2008; Roy et al. 2014).

Of the salt tolerance mechanisms listed above, shoot ion-independent tolerance has received relatively little research attention. Using non-destructive plant imaging technologies, genetic variation has been observed for shoot tolerance mechanisms not related to shoot ion concentration in barley (*Hordeum vulgare*) (Tilbrook et al. 2017), bread wheat (Takahashi et al. 2015), chickpea (Atieno et al. 2017), durum wheat (Sirault et al. 2009), rice (Al-Tamimi et al. 2016; Campbell et al. 2015) and *Triticum monococcum* (Rajendran et al. 2009). In rice, a genome-wide association study (GWAS) detected marker–trait associations with shoot ion-independent tolerance, with a number of candidate genes suggested (Al-Tamimi et al. 2016). However, to date, no genes for shoot ion-independent tolerance have been identified in bread wheat.

Here we screened a bread wheat doubled haploid (DH) mapping population (Excalibur × Kukri) in the glasshouse under control and salinity treatments to identify novel QTL for a number of salinity tolerance sub-traits. Promising QTL from the glasshouse trials were then validated in the field by testing a set of Excalibur × Kukri DH lines across a range of salinity concentrations at multiple field trial sites for 2 years.

## Materials and methods

### Plant materials

A DH mapping population of Excalibur × Kukri was developed from the F_1_ generation of a cross between single plants of two Australian wheat cultivars mixed haplotypes Excalibur (RAC177/Uniculm492//RAC311S) and Kukri (76ECN44/76ECN36//RAC549/Madden/6*RAC177). The Excalibur parent used in this population was a single plant known as Excalibur-198. This population was previously used for mapping loci affecting ion accumulation (Edwards 2012; Shavrukov et al. 2011), nematode resistance (Jayatilake et al. 2013) and metabolite abundance (Hill et al. 2013, 2015). Excalibur is a drought-tolerant wheat cultivar and has high yields in the wheat belt of South Australia but produces low-quality grain and is susceptible to rust. Kukri is a hard-white wheat cultivar that has low drought and heat tolerance but produces excellent quality grain and is resistant to rust (Izanloo et al. 2008). The Excalibur and Kukri parents, along with 212 DH lines, were used for controlled glasshouse phenotyping. A set of 20 DH lines from this population with high and low shoot ion-independent tolerance (maintenance of growth during early phase (1–5 days) of salt treatment) and 18 lines with high and low ion-dependent tolerance (leaf Na^+^ exclusion) along with both parents were used for field trials (Supplementary Table 1). Three plants from each of 44 Australian bread wheat cultivars and one landrace were genotyped for a single nucleotide polymorphism (SNP) (X2279012.58AC) that was found to be linked with a shoot ion-independent tolerance QTL on chromosome 7A (*QG*_*(1***–***5)*_*.asl*-*7A*). This genotyping was conducted using two forward primers 5′-GAAGGTGACCAAGTTCATGCTGGGGCGATAAGGACGCGGA-3′ and 5′-GAAGGTCGGAGTCAACGGATTGGGCGATAAGGACGCGGC-3′ (including the underlined FAM and VIC tails, respectively) and a common primer 5′-CCTTACGCATGTAAGCATTTCCCGAA-3′ in a Kompetitive Allele Specific PCR (KASP™) assay (www.lgcgroup.com/our-science/genomics-solutions/genotyping/kasp-genotyping-chemistry).

### Non-destructive glasshouse-based phenotyping

A phenotyping experiment was conducted from late winter to early spring (29 August to 26 September 2011) using a fully automated conveyer system containing carts for pots of plants within a temperature-controlled Smarthouse (The Plant Accelerator^®^, Adelaide, Australia; longitude: 138.64, latitude: − 34.97). The experiment was a partially replicated design with 20% of the population duplicated and the parents (Excalibur and Kukri) replicated six times. Seeds of the Excalibur and Kukri parents along with 212 Excalibur × Kukri DH lines were imbibed in reverse osmosis (RO) water at room temperature for 2 h and placed in the dark at 4 °C for 48 h. Four seeds were sown in a white pot (19.5 cm height × 15 cm diameter, Berry Plastics Corporation, Evansville, USA) filled with 2.6 kg of soil potting mixture (50% (v/v) University of California mix, 35% (v/v) peat mix and 15% (v/v) clay loam soil from Angle Vale, South Australia, Australia. After sowing, plants were grown under natural light with temperatures at 22 °C days and 15 °C night. All the pots were arranged according to a split-plot design, which was constructed using DiGGer (Coombes 2009) and dae (Brien 2015b). In the design (multiline experiment 3), two carts/pots in the same position in adjacent lanes form a main plot (Brien et al. 2013). The main-plot design is a blocked, nearly trend-free, partially replicated design that assigns lines to main plots; the subplot design randomizes conditions (control, salt) to the two carts in each main plot.

Plants were thinned to one seedling per pot at the second leaf stage. At the emergence of the third leaf (14 days after planting), pots were loaded into carts on the Smarthouse conveyer system maintaining the split-plot design. Seedlings were watered to weight every second day to maintain the soil water content at 17% gravimetric water content. At emergence of the fourth leaf (18 days after planting), NaCl solution was added to the saucer to reach a final concentration of 100 mM NaCl in the soil solution. Pots were not watered again until the soil water content went below 17% (g/g). Each pot was then watered automatically to maintain the soil water content at 17% (g/g) and salinity at 100 mM NaCl. This was achieved by the electronic conveyer system weighing the pot every second day on industrial scales (Bizerba, Balingen, Germany) and applying the appropriate amount of RO water.

The LemnaTec Scanalyser 3D (LemnaTec GmbH, Aachen, Germany) at The Plant Accelerator^®^ was used for non-destructive measurements of plant growth. Three 5 megapixel red–green–blue (RGB) images of each plant were recorded every sampling day, one from the top view and two from the side view at a 90^°^ rotation to each other (Golzarian et al. 2011; Rajendran et al. 2009; Takahashi et al. 2015). Imaging started 4 days before salt treatment and continued for 10 days after salt application. After 10 days of salt treatment, the fully expanded fourth leaf was sampled for ion concentration (Na^+^ and K^+^) and the youngest fully emerged leaf was sampled for DNA extraction and genotyping.

### Shoot ion-independent tolerance calculation

The total shoot pixel area derived from the three RGB images were used to calculate the projected shoot area (PSA, kpixels) of each plant (Golzarian et al. 2011; Rajendran et al. 2009). A linear correlation between shoot biomass and PSA has been observed in *Triticum monococcum* (Golzarian et al. 2011) and bread wheat (Takahashi et al. 2015). To calculate the growth rate of an individual plant, a cubic smoothing spline was fitted to the PSA for the plant as measured over time, using the R function smooth.splines with degrees of freedom (*df*) set to 4 (Al-Tamimi et al. 2016). The relative growth rate (RGR) was calculated for each plant for each day it was imaged as RGR $\left( {{\text{kpixels}}\,\,{\text{d}}^{ - 1} \,\,{\text{kpixels}}^{ - 1} } \right) = \left( {{\text{dln}}\left( A \right)/{\text{d}}t} \right),$ where *A* is the projected shoot area, *d*ln(*A*) is the difference in logarithm projected shoot area over the time period d*t*). Also, the RGR was calculated for each plant for the intervals 1–5 days after salt treatment. The ability to maintain growth after salt application (1–5 days) was calculated for each DH line or parent as the ratio of the RGR for the salt-treated plant divided by the RGR for the control plant.

### Statistical analysis of imaging data

In the glasshouse, the RGR for the intervals 1–5 days after salt treatment were analysed by fitting mixed models using asreml (Butler et al. 2009) and asremlPlus (Brien 2015a). The maximal model included fixed effects for genotype, treatment and their interaction, random components for zones, main plots and residual variation and terms for curved trend and deviations from trend for lanes and positions. Random terms were tested for significance and removed if not significant. The shoot ion-independent tolerance was analysed using a model with fixed genotype effects and random zone and residual variation.

### Measurements of leaf Na^+^ and K^+^ concentrations

The fourth leaf, which was fully expanded under salt treatment, was harvested after 10 days of salt treatment. Leaf fresh weight was measured and the samples dried in an oven for 2 days at 65 °C before the dry weight was recorded. Dried leaves were digested in 10 mL of 1% (v/v) HNO_3_ at 85 °C for 4 h in a 54-well Hotblock (Environmental Express, Mount Pleasant, SC, USA). The concentration of Na^+^ and K^+^ was measured using a flame photometer (Model 420 Sherwood, Cambridge, UK).

### Field-based phenotyping

Field trials were conducted at Cunderdin, Western Australia (WA) (2014 and 2015), Whitwarta, South Australia (SA) (2014 and 2015) and Coomandook (SA) (2015). Each trial consisted of a low and high salinity site ranging from 100 to 500 m apart within the same paddock (Table 1). The total area of each site was up to 0.7 ha with the Excalibur × Kukri experiment comprising 20% of this area. Growing season rainfall and temperature values (Supplementary Table 2) and rainfall and temperature values for each month (Supplementary Figure 1) were obtained from the Bureau of Meteorology (BOM) for each site. For each trial, the plots were arranged in a rectangular array of rows and ranges (12 ranges by 10 rows) and a spatially optimized row–column design was used to assign three replicates of each of 48 lines and the two parents to the plots. The designs were obtained using the R statistical software environment (R Development Core Team 2014) with the DiGGer package (Coombes 2009). In SA, 70 g of seed was sown per plot for an initial plot size of 1.32 m × 7 m at sowing and a final plot size of 1.32 m × 5 m reduced by herbicide application prior to stem elongation. In WA, 50 g of seed was sown per plot with an initial plot size of 1.32 m × 6 m at sowing and a final plot size of 1.32 m × 4 m. Standard agronomic practices were used including pest and disease treatments, weed control and fertilizer applications. To reduce potential crown rot issues, the seed sown at Cunderdin in 2014 was treated with EverGol^®^ Prime (Penflufen: 240 g/L active ingredient) at a rate of 6 mL of EverGol Prime per kg of seed.

**Table 1:** The apparent electrical conductivity (EC_a_, mS/m) measured in the horizontal mode at 0–50 cm (EC_ah_) and vertical mode at 0–100 cm (EC_av_) in October (Whitwarta and Coomandook), August (Cunderdin in 2014) and September (Cunderdin in 2015), the calculated salinity of the soil solution (mM) and soil electrical conductivity (dS/m) at 0–25 and 25–50 cm

Year	Location	Site	EM38 value 0–50 cm (EC_ah_) (mS/m)		EM38 value 0–100 cm (EC_av_) (mS/m)		Soil solution (mM) 0–25 cm		Soil solution (mM) 25–50 cm		Soil EC_1:5_ (dS/m) 0–25 cm		Soil EC_1:5_ (dS/m) 25–50 cm		*n*
			Mean	SEM	Mean	SEM	Mean	SEM	Mean	SEM	Mean	SEM	Mean	SEM	
2014	Whitwarta	Low salt	53	0.4	80	0.5	113	0.03	104	0.8	0.13	0.0004	0.18	0.0019	504
		High salt	100	0.3	157	0.4	141	0.7	179	0.7	0.18	0.0008	0.34	0.0030	504
	Cunderdin	Low salt	nd	nd	nd	nd	nd	nd	nd	nd	nd	nd	nd	nd	nd
		High salt	221	1.6	276	1.3	445	11	646	12	0.79	0.020	1.46	0.0235	585
2015	Coomandook	Low salt	136	0.5	90	0.4	43	0.1	44	0.1	0.03	0.0003	0.06	0.0007	504
		High salt	151	1.0	113	1.0	308	1.0	244	1.0	0.45	0.0100	0.57	0.0130	396
	Whitwarta	Low salt	60	0.7	41	0.4	33	0.2	40	0.03	0.09	0.0003	0.11	0.0001	504
		High salt	175	1.3	124	1.1	70	1.6	119	4.5	0.27	0.0098	0.40	0.0113	504
	Cunderdin	Low salt	70	1.0	98	1.2	61	0.5	90	1.5	0.11	0.0020	0.25	0.0060	585
		High salt	216	1.5	261	1.3	268	5.0	349	4.3	0.50	0.0141	1.02	0.0157	598

Values derived from calibration curves between measured soil EC_1:5_ and salinity of the soil solution with EM38 values at 0–50 cm for Cunderdin and 0–100 cm for Whitwarta and Coomandook

*n* the number of plots tested, *nd* no data

In July and October of each year, the apparent electrical conductivity (EC_a;_ mS/m) at two soil depths (0–50 and 0–100 cm) of each low and high salinity trial was measured using a handheld EM38 Geonics device (Cunderdin only) or a vehicle-fitted EM38 Geonics device (Precision Ag Services, Minalton, Australia) (Whitwarta and Coomandook), except in 2014, where the low salinity site at Cunderdin was not measured (Supplementary Figure 2). At Whitwarta and Coomandook, based on the EM38 maps at 0–100 cm, soil was collected from a depth of 0–25 and 25–50 cm in regions of the trial site corresponding to EM38 values ranging every 5th percentile (low and high salt) from the minimum to maximum recorded EM38 value for each trial area. At Cunderdin, based on the EM38 maps at 0–50 cm, soil was collected from 0–25 and 25–50 cm at locations of the trial at every 5th percentile (high salt 2014), every 6.7th percentile (low salt 2015) and every 3.5th percentile (high salt 2015). Soil gravimetric water content (g/g) was determined by recording the fresh weight and weight after drying in an oven at 80 °C for 3 days. Soil electrical conductivity (EC_1:5_) and pH (water) were measured using a CyberScan PC510 m (Eutech Instruments, Thermo Fisher Scientific Inc., Waltham, MA, USA) in a 1:5 (soil:water) extract after shaking for 1 h and settling for 30 min. The concentration of Na^+^, K^+^ and Clˉ was measured in the soil:water extract using a flame photometer (Model 420, Sherwood Scientific, Cambridge, UK) and chloride meter (Model 926, Sherwood Scientific) for the Whitwarta and Coomandook trials. The salinity of soil solution (mM) was calculated using the following formula: EC_1:5_ (dS/m) × 5000/soil water content (g/g) (Setter et al. 2016). By fitting a line of best fit to a plot of the EM38 values versus the measured EC_1:5_ value including the *R*^2^ value and a regression analysis (*p* value) (Supplementary Table 3), an estimation of the EC_1:5_ for each plot at 0–25 cm and 25–50 cm was derived from the measured EM38 value for each plot (Table 1).

At Z60, ten penultimate flag leaves were randomly collected from each plot in both low and high salinity trials, except for Cunderdin in 2014 where leaf tissue was collected from the high salinity trial only. The leaf tissue was oven-dried at 80 °C for 3 days, ground by hand and subsampled (around 0.14 g per sample) into a 50-mL tube. Subsampled leaf tissue was digested in 10 mL of 1% HNO_3_ (v/v) at 85 °C for 4 h in a HotBlock (Environmental Express, Mount Pleasant, SC, USA). The leaf Na^+^ and K^+^ concentration was measured using a flame photometer (Model 420, Sherwood Scientific) (Shavrukov et al. 2010). If applicable to the trial site, scoring for plant establishment (plants/m^2^), plant lodging (% of plot), extent of weeds (% of plot) and frost damage (% of plot) was recorded and used in the plot yield covariate analysis where necessary. Total plot grain yield was recorded (t/ha) for all trials and the 1000 grain weight recorded for the Cunderdin trials only.

For field data analysis, a mixed model analysis for each trait was performed using ASReml-R (Butler et al. 2009) and asremlPlus (Brien 2015a), with the mixed model including (1) random row and column terms, (2) first-order autocorrelation for rows and columns, (3) a fixed effect term for lines and (4) covariates as described in Supplementary Table 4 and that included EM38 and EC_1:5_ measurements. REML ratio tests were carried out for both the rows and columns autocorrelation and when not significantly removed from the model.

### DNA extraction and KASP™ genotyping

Genomic DNA was extracted from the youngest fully expanded leaf using the phenol/chloroform method (Pallotta et al. 2000; Rogowsky et al. 1991). DNA was quantified using a NanoDrop 1000 spectrophotometer (Thermo Fisher Scientific, Wilmington, DE) and diluted to 1 ng/μl for use in the KASP™ assay (www.lgcgroup.com/our-science/genomics-solutions/genotyping/kasp-genotyping-chemistry).

### Construction of Excalibur × Kukri DH genetic map

A genetic linkage map of the Excalibur × Kukri DH population with 233 lines was previously constructed by Edwards (2012) and updated by Jayatilake et al. (2013) with new markers for 182 lines. Additional 12 KASP™ markers were developed using sequencing data from the 9 K Wheat Illumina Infinium iSelect genotyping array (Cavanagh et al. 2013); KASP™ markers from the Avalon × Cadenza genetic linkage map (Allen et al. 2013) and CerealDB database (http://www.cerealsdb.uk.net/cerealgenomics/CerealsDB/kasp_mapped_snps.php/) were added to the existing Excalibur × Kukri DH genetic map using MapManager QTXb20 (Manly et al. 2001). Recombination frequencies were converted to cM using the Kosambi mapping function (Kosambi 1943), and the marker order was optimized using the recombination counting and ordering (RECORD) program (Van Os et al. 2005).

A second genetic linkage map for the Excalibur × Kukri DH population was developed using genotyping by sequencing (GBS) conducted by Diversity Arrays Technology (DArT) Pty Ltd (Canberra, Australia) using the DArTseq assay (http://www.diversityarrays.com/). The two genetic linkage maps were combined together using the combineMap function of R/ASMap (version 0.4-7) (Taylor 2015). The combined map was curated by removing markers having > 20% missing data and significant segregation distortion (> 0.6 or < 0.4 (*p* = 0.05). Similarly, the lines that showed a high observed count of crossovers (threshold = 100) were also removed. The final integrated linkage map consisted of 155 individuals with 3503 markers assigned to 27 linkage groups, representing all chromosomes, except 4B, with a total length of 2707 cM, (1140, 961 and 606 cM for the A, B and D genomes, respectively) (Supplementary Table 5). Despite numerous attempts, and adjustment of parameters in the mapping programs, it was not possible to identify any polymorphisms on chromosome 4B.

### Quantitative trait loci (QTL) analysis

Using WinQTLCart version 2.5 (Wang et al. 2007) (Model 6 standard analysis with 5 control markers and a window size of 10 cM), composite interval mapping (Zeng 1994) was performed on the 212 lines phenotyped in 2011 using genotyping data obtained in 2014 of 129 lines (Jayatilake et al. 2013) (Supplementary Table 6) (Wang et al. 2007). Log of odds (LOD) thresholds were determined with 1000 permutations (Churchill and Doerge 1994) at a 1 cM walk (*p* = 0.05). Significant QTL were summarized with their position on a linkage group, LOD score, magnitude and directions of their estimated additive effects and their contribution to the genetic variance. The notation for individual QTL followed the recommended format for wheat: *Qphenotype.lab*-*chromosome.QTL number* with ‘asl’ signifying ‘Adelaide Salt Lab’. The *G*_*(1***–***5)*_ refers to shoot ion-independent growth calculated over 1–5 days, *CRGR* the relative growth rate under control condition, *Na* the leaf Na^+^ concentration and *K* the leaf K^+^ concentration. Unique QTL were defined as those where a subset of correlated traits mapped within 15 cM of one another (Sewell et al. 2000, 2002).

### Determining the effect of allele on phenotype in the field

To determine the effect of the salinity tolerance alleles on yield and performance in the field, the genotypic and field trials phenotypic data were aligned. For the Excalibur × Kukri DH lines selected based on their glasshouse phenotype (high or low shoot ion-independent tolerance and/or low and high leaf Na^+^ concentration), the statistically adjusted mean value for the phenotypic traits measured at each field trial (leaf Na^+^ and K^+^ concentrations and grain yield) was aligned with the genotypic data of each line. For each significant marker of a QTL detected in the glasshouse experiment, the DH lines were sorted by their allele (E = Excalibur—or K = Kukri). The performance of field grown lines, with either the Excalibur or Kukri alleles, was assessed based on whether their phenotype was consistent with that of the parents observed in the glasshouse. The mean value for each trait for lines grouped with either the E or K allele was calculated and the % increase or decrease from that allele for each trait was determined.

### Physical mapping of the QTL

To generate a list of genes underlying specific QTL, all the markers (Supplementary Table 7) within the detected region, that were up to 2 LOD drops from the maximum likelihood value of selected QTL, were physically positioned on the wheat genome sequence. The appropriate SNP-bearing sequences were probed to the entire bread wheat NRGene genome assembly version 1.0 (International Wheat Genome Sequencing Consortium, https://wheat-urgi.versailles.inra.fr/Seq-Repository/Assemblies) using an in-house BLAST portal. The limit of acceptance of assignment was based on the query sequence having a cumulative identity percentage of similarity (> 96%) and a cumulative alignment length percentage of matches (90–100%) to the hit from the sequence database. The wheat genome scaffolds containing the marker, as well as the genome scaffolds bridging the region between the two flanking markers, were retrieved from the BLAST searches and used to find expressed genes on the scaffolds, using POTAGE (PopSeq Ordered *Triticum aestivum* Gene Expression) (Suchecki et al. 2017) and an in-house tool, DAWN (diversity among wheat genomes), developed at the Australian Centre for Plant Functional Genomics.

DAWN is a bioinformatics tool, which utilizes the International Wheat Genome Sequencing Consortium (IWGSC; www.wheatgenome.org/) bread wheat scaffolds version 3, pseudomolecule assembly version 0.4 (https://wheat-urgi.versailles.inra.fr/) to aggregate a wide range of bread wheat genomic and transcriptomic data. This includes a Bioplatforms Australia (BPA) whole-genome sequencing (WGS) dataset of 16 Australian wheat cultivars (Edwards et al. 2012), including Excalibur and Kukri. DAWN includes the location of high confidence gene predictions from International Wheat Genome Sequencing Consortium (2014) as well as RNA-Seq expression data from five wheat tissues (root, leaf, stem, spike and grain), taken at three developmental stages (seedling, vegetative and flowering) under normal growth conditions (https://urgi.versailles.inra.fr/files/RNASeqWheat/) (Choulet et al. 2014; Rustenholz et al. 2011). POTAGE integrates map location with gene expression and inferred functional annotation and visualizes these data through a web browser interface (Suchecki et al. 2017).

## Results

### Glasshouse phenotyping

**Fig. 1:**
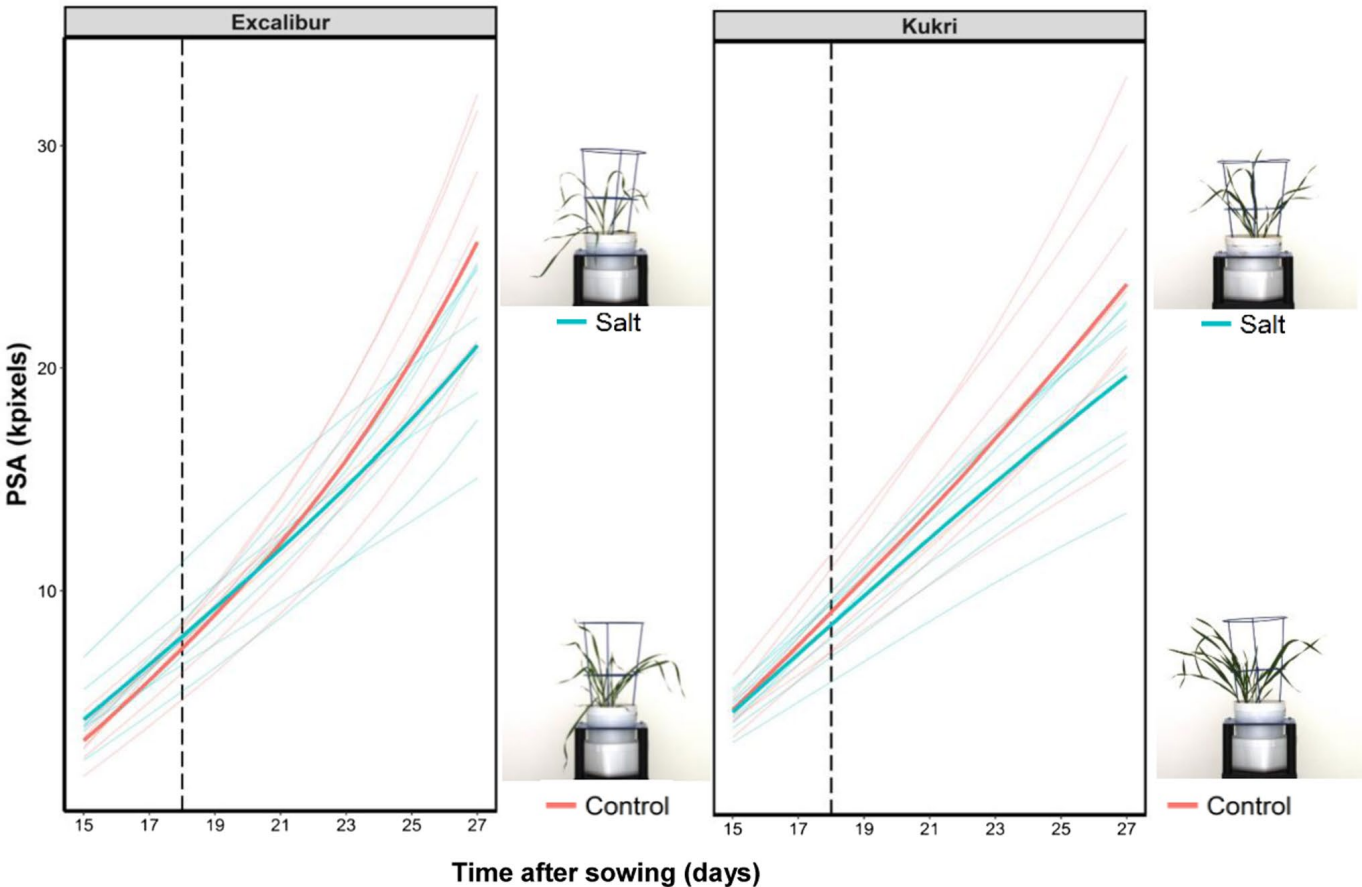
The projected shoot area (PSA) of Excalibur and Kukri plants under control (red line) and salt stress (100 mM NaCl, blue line) from days 15 to 27 after sowing. The RGB side-view images of plants showing the difference between growth of control and salt-treated plants. The vertical dotted line indicates the time point of salt application. Bold red and blue lines represent the mean values while the other ones are the individual replicates

**Fig. 2:**
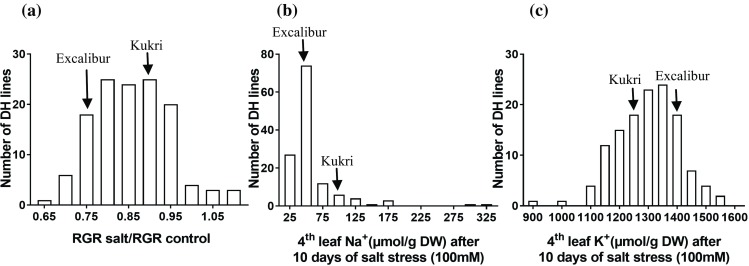
Frequency distribution of **a** shoot ion-independent tolerance (days 1–5) and fourth leaf **b** Na^+^ accumulation (µmol/g DW) and **c** K^+^ accumulation (µmol/g DW) measured after 10 days of salt stress (100 mM) in the Excalibur × Kukri DH population. Arrows indicate the trait mean for parents, Excalibur and Kukri

The parents Excalibur and Kukri had on average 30 and 24% reduction in PSA, respectively, after 10 days of 100 mM NaCl when compared to control plants (Fig. 1). The ratio of relative growth rates (RGR_salt_/RGR_control_) after 5 days of salt treatment was used to determine the ability of each line to maintain growth. The range of shoot ion-independent tolerance values observed over the first 5 days of salt treatment in the DH population was from 0.66 to 1.09. Kukri and Excalibur had relative growth rates of 0.88 and 0.78, respectively (Fig. 2a).

The fourth leaf ion concentration measured in the DH population had a skewed distribution for Na^+^ (µmol/g DW) accumulation, with 88 lines having a lower Na^+^ concentration than either parent. The mean fourth leaf Na^+^ accumulation for Excalibur was 56 ± 9.02 µmol Na^+^/g DW and 110 ± 27.63 µmol Na^+^/g DW for Kukri with a range of 25–315 µmol Na^+^/g DW in the DH lines (Fig. 2b). A normal distribution for K^+^ accumulation was observed (Fig. 2c), with Excalibur accumulating 1411 ± 51 µmol K^+^/g DW and Kukri accumulating 1275 ± 36 µmol K^+^/g DW. The DH lines had a range of 917–1566 µmol K^+^/g DW.

### QTL for salinity tolerance traits in the glasshouse

**Fig. 3:**
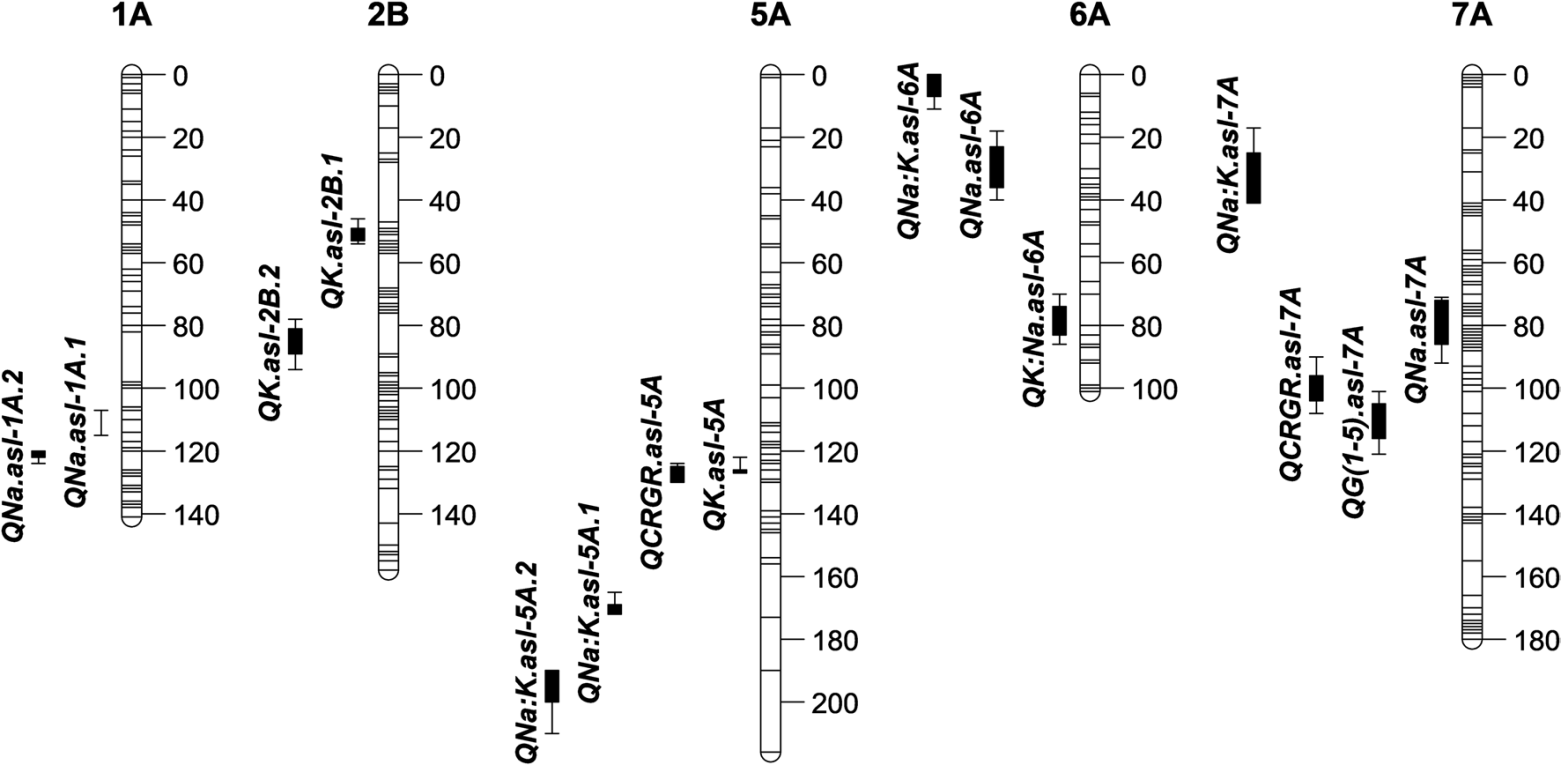
QTL positions for observed traits in the Excalibur × Kukri DH population under control and salt stress (100 mM NaCl for 10 days). The vertical QTL bars represent the 1 and 2 LOD drops from the maximum likelihood value. QTL and their positions are indicated: for Growth_(1–5)_ (*QG*_*(1***–***5)*_*.asl*-*7A*), RGR (*QRGR.asl*-*5A* and *QRGR*-*7A*), 4th leaf Na^+^ accumulation (µmol/g DW) (*QNa.asl*-*1A.1*, *QNa.asl*-*1A.2*, *QNa.asl*-*6A* and *QNa.asl*-*7A*), K^+^ accumulation (µmol/g DW) (*QK.asl*-*2B.1*, *QK.asl*-*2B.2* and *QK.asl*-*5A*), Na^+^:K^+^ (DW) (*QNa:K.asl*-*5A.1*, *QNa:K.asl*-*5A.2*, *QNa:K.asl*-*6A*, *QNa:K.asl*-*7A*) and K^+^:Na^+^ (DW) (*QK:Na.asl*-*6A*). RGR = relative growth rate (days 1–5), Growth_(1–5)_ = RGR of plants between 1 and 5 days after salt stress (RGR salt/RGR control), respectively

Under both salt and control treatments, 15 QTL were detected at 10 unique loci with LOD scores ranging between 2.6 and 12.9 (Table 2, Fig. 3). A range of phenotypic variation and additive effects were observed for the individual QTL (Table 2).

**Table 2:** QTL for salt tolerance sub-traits in the Excalibur × Kukri DH mapping population under control and salt stress (100 mM NaCl for 10 days). Trait name, QTL name, treatment, chromosome (Chr), position on chromosome, most significant marker, LOD score, additive effect and phenotypic variation (*R*^2^) explained by the QTL (% variation) are shown

Trait	QTL	Treatment	Chr	Position	Marker	LOD	Additive	*R* ^2^
Growth_(1**–**5)_	*QG*_*(1***–***5)*_*.asl*-*7A*	Salt/control	7A	112.31	X2279012.58AC	5.0	0.035	14.1
RGR	*QCRGR.asl*-*5A*	Control	5A	126.21	X1264710.28CG	12.9	0.006	29.8
RGR	*QCRGR.asl*-*7A*	Control	7A	99.41	X1116135.61GA	3.6	− 0.004	7.0
Na^+^ µmol/g DW	*QNa.asl*-*7A*	Salt	7A	75.01	wmc0017	3.7	− 15.7	11.3
Na^+^ µmol/g DW	*QNa.asl*-*1A.1*	Control	1A	106.61	X2264210.64CG	3.3	14.5	10.1
Na^+^ µmol/g DW	*QNa.asl*-*1A.2*	Control	1A	120.41	X1125323.58TG	5.9	− 16.6	18.2
Na^+^ µmol/g DW	*QNa.asl*-*6A*	Control	6A	29.91	X1127808.37CG	2.7	4.6	4.9
K^+^ µmol/g DW	*QK.asl*-*2B.1*	Salt	2B	52.61	X1103701.44AG	3.8	39.7	8.1
K^+^ µmol/g DW	*QK.asl*-*2B.2*	Salt	2B	87.81	X1022175.20CT	6.1	− 50.0	12.1
K^+^ µmol/g DW	*QK.asl*-*5A*	Salt	5A	125.51	Vrn-A1	12.9	57.4	28.2
Na^+^: K^+^ DW	*QNa:K.asl*-*5A.1*	Control	5A	172.21	X1130301.5CT	5.0	− 0.020	13.8
Na^+^: K^+^ DW	*QNa:K.asl*-*5A.2*	Control	5A	189.81	X1083587.10GA	3.0	0.015	8.0
Na^+^: K^+^ DW	*QNa:K.asl*-*6A*	Control	6A	0.01	X1397091.19CT	3.8	0.012	10.1
Na^+^: K^+^ DW	*QNa:K.asl*-*7A*	Control	7A	39.31	X1193500.27AT	2.6	− 0.010	6.6
K^+^: Na^+^ DW	*QK:Na.asl*-*6A*	Salt	6A	80.31	X3023657.26CG	3.6	− 3.4	10.1

RGR = relative growth rate (days 1–5), Growth (1**–**5) = RGR of plants between 1 and 5 days after salt stress (RGR salt/RGR control)

A single QTL for the ability to maintain growth under salinity was mapped on chromosome 7A (*QG*_*(1***–***5)*_*.asl*-*7A*) with a LOD score of 5.0. It accounted for 14.1% of the phenotypic variation and had a positive effect from Excalibur (Table 2).

In the control treatment, two QTL were detected for RGR (1–5) on chromosomes 5A (*QCRGR.asl*-*5A*) and 7A (*QCRGR.asl*-*7A*) with LOD scores of 12.9 and 3.6, respectively (Table 2). These two QTL accounted for 29.8 and 7% of the phenotypic variation and the additive effect attributed with these QTL was small. These two QTL were not observed in the salt-treated plants.

A total of four QTL were detected for fourth leaf Na^+^ concentration including one QTL in salt-treated plants and three in the control treatment (Table 2). The QTL for Na^+^ concentration in the fourth leaf of salt-treated plants was located on chromosome 7A (*QNa.asl*-*7A*), with a LOD of 3.7 and 11.3% total phenotypic variation. The additive effect was − 15.7, indicating that Excalibur is a better excluder of Na^+^ than Kukri. The three QTL detected in the control treatment were mapped on chromosomes 1A (*QNa.asl*-*1A.1* and *QNa.asl*-*1A.2*) and 6A (*QNa.asl*-*6A*) with LOD scores of 3.3, 5.9 and 2.7, respectively. The phenotypic variances for *QNa.asl*-*1A.1*, *QNa.asl*-*1A.2* and *QNa.asl*-*6A* were 10.1, 18.2 and 4.9%, respectively, with additive effects of 14.5, − 16.6 and 4.6, respectively. This suggests the Kukri alleles at *QNa.asl*-*1A.1* and *QNa.asl*-*6A*, and the Excalibur allele at *QNa.asl*-*1A.2* are linked to Na^+^ exclusion.

For fourth leaf K^+^ concentration, three QTL were detected in the salt-treated plants: on chromosomes 2B (*QK.asl*-*2B.1*and *QK.asl*-*2B.2*) and chromosome 5A (*QK.asl*-*5A*), with LOD scores of 3.8, 6.1 and 12.9, respectively. The phenotypic variances explained by these QTL were 8.1, 12.1 and 28.2%, respectively (Table 2). One of the QTL on chromosome 2B (*QK.asl*-*2B.2*) had the additive effect (− 50.0) being inherited from Kukri, while the second QTL on chromosome 2B (*QK.asl*-*2B.1*) and the QTL on chromosome 5A (*QK.asl*-*5A*) showed the additive effect (39.7 and 57.4) that was inherited from Excalibur.

For the fourth leaf Na^+^:K^+^ DW in the control treatment, four QTL were mapped (Table 2): two on chromosome 5A (*QNa:K.asl*-*5A.1* and *QNa:K.asl*-*5A.2*), one on chromosome 6A (*QNa:K.asl*-*6A*) and one on chromosome 7A (*QNa:K.asl*-*7A*). These had LOD scores ranging from 2.6 to 5.0 and explained between 6.6 and 13.8% of the phenotypic variance. One significant QTL in the salt treatment was detected for K^+^:Na^+^ DW on chromosome 6A (*QK:Na.asl*-*6A*) with a LOD score of 3.6, explaining 10.1% of the phenotypic variation. At this QTL, the positive effect (3.4) was inherited from Kukri.

### Soil electrical conductivity at the field trial sites

There was variation in apparent electrical conductivity (EC_a_) at all field sites (Table 1 and Supplementary Figure 2). At each field location in all years, the mean EM38 values at 0–50 and 0–100 cm were less in the low salt sites compared to the high salt sites (Table 1). Between field locations, Cunderdin 2014 and 2015 high salt sites had the highest EC_a_ values at 0–50 and 0–100 cm (Table 1). The low salt site at Coomandook in 2015 at 0–50 cm had a lower EC_a_ than the high salt site at the same location but had a higher apparent conductivity than either of the low and high salt sites at Whitwarta in 2014 and the low salt sites at Whitwarta and Cunderdin in 2015 (Table 1).

The soil electrical conductivity (EC_1:5_) and salinity of the soil solution confirmed the presence of low and high salinity sites at each location (Table 1). Average trial plot values of salinity in the soil solution ranged from 33 to 445 mM at 0–25 cm and 40 to 646 mM at 25–50 cm (Table 1). Sites with the highest salinity of the soil solution were Cunderdin high salt in 2014 and 2015, Coomandook high salt in 2015 and Whitwarta high salt in 2014. Average trial plot soil EC_1:5_ values ranged from 0.03–0.79 dS/m at 0–25 cm to 0.06–1.46 dS/m at 25–50 cm (Table 1). Locations with the high EC_1:5_ were Cunderdin high salt in 2014 and 2015 and Coomandook in 2015 and Whitwarta high salt sites in 2014 and 2015 (Table 1).

### Effect of alleles on Na^+^ and K^+^ accumulation

For the Na^+^ accumulation QTL on chromosome 7A (*QNa.asl*-*7A*), lines with the allele from the Na^+^ exclusion parent (Excalibur) had a decrease in Na^+^ at six out of nine sites, consistent with QTL detected in the glasshouse (Table 3). The most significant amount of Na^+^ exclusion was observed at the low salt site in Cunderdin 2015, where 33.6% more Na^+^ was excluded compared to the lines carrying the Na^+^ accumulation allele. The difference observed in Na^+^ exclusion at the other three sites ranged from 0.8 to 13.3% (Table 3).

**Table 3:** The percentage difference in field leaf Na^+^ and K^+^ (µmol/g DW) concentration and % difference in grain yield linked to the Na^+^ and K^+^ alleles of interest. The table shows the QTL name, and the beneficial alleles for that trait as identified in the glasshouse (E = Excalibur, K = Kukri), for locations at Whitwarta, Cunderdin, Coomandook in 2014 and/or 2015

Year	Location	Site	Mean EM38 value, 0–50 cm (mS/m)	% Difference in ion accumulation in the field					% Difference in grain yield in the field					
				Leaf Na^+^	Leaf K^+^	Leaf K^+^	Leaf K^+^	Leaf K^+^:Na^+^						
				*QNa.asl*-*7A* (Allele = E)	*QK.asl*-*2B.1* (Allele = E)	*QK.asl*-*2B.2* (Allele = K)	*QK.asl*-*5A* (Allele = E)	*QK:Na.asl*-*6A* (Allele = K)	*QG(1*_**–**_*5).asl*-*7A* (Allele=E*)*	*QNa.asl*-*7A* (Allele = E)	*QK.asl*-*2B.1* (Allele = E)	*QK.asl*-*2B.2* (Allele = K)	*QK.asl*-*5A* (Allele = E)	*QK:Na.asl*-*6A* (Allele = K)
2014	Whitwarta	Low salt	53 ± 0.4	16.4	**0.9**	− 0.1	− 5.6	**8.5**	**4.9**	**4.0**	− 1.8	**3.0**	− 3.5	− 4.7
		High salt	100 ± 0.3	11.5	− 11.5	**13.9**	− 5.1	**15.0**	**4.6**	**6.9**	**0.6**	**1.6**	− 5.1	− 4.9
	Cunderdin	Low salt	nd	nd	nd	nd	nd	nd	− 1.8	− 1.3	− 2.1	**2.3**	− 0.4	− 1.9
		High salt	221 ± 1.6	− **4.27**	− 0.4	**1.5**	− 9.2	− 9.8	− 2.3	− 2.6	− 5.4	**3.7**	**3.7**	**2.0**
2015	Coomandook	Low salt	136 ± 0.5	− **13.3**	**4.6**	− 2.2	− 0.7	**1.0**	− 11.5	− 12.5	− 1.9	**3.1**	**18.7**	**15.3**
		High salt	151 ± 1.0	− **12.4**	− 2.4	**4.7**	− 4.6	− 24.6	− 0.8	− 4.7	− 7.1	**13.1**	− 0.4	− 5.4
	Cunderdin	Low salt	70 ± 1.0	− **33.6**	**1.5**	− 1.8	− 1.4	**10.6**	− 3.3	− 5.7	− 1.8	**2.4**	**5.1**	− 0.7
		High salt	216 ± 1.5	7.4	**1.3**	**0.8**	− 1.0	**9.5**	− 0.7	− 1.8	**1.2**	**1.1**	**12.5**	**15.4**
	Whitwarta	Low salt	60 ± 0.7	− **13.2**	− 3.0	**2.3**	**0.8**	**11.5**	− 4.5	**12.0**	**9.4**	**4.6**	− 7.8	− 9.2
		High salt	175 ± 1.3	− **0.8**	− 9.4	**7.9**	− 0.4	− 0.1	− 13.3	− 1.3	**28.0**	**3.2**	− 7.3	**1.0**

The mean EM38 value at 0–50 cm is shown for each site, along with the % increase or decrease in the grain yield associated with the alleles of interest. Sites with the desired response (i.e. sodium exclusion, K^+^ accumulation or high grain yield) with the allele of interest are highlighted in bold

*nd * no data

Of the four K^+^ accumulation QTL, two (*QK.asl*-*2B.2* and *QK:Na.asl*-*6A*) were observed to have the additive effect from the Kukri parent and showed increased accumulation at six out of nine sites (Table 3). For *QK.asl*-*2B.2*, lines with the Kukri allele accumulated 13.9 and 7.9% more K^+^, respectively, than other lines in the 2014 and 2015 high salt sites at Whitwarta, and lines with this Kukri allele also had K^+^ accumulation (ranging from 0.8 to 4.7%) at four other sites (Table 3). At *QK:Na.asl*-*6A*, the highest K^+^ accumulation effect was observed at the high salt site at Whitwarta in 2014 (15.0%) while at other sites this ranged from 1 to 11.5%. For the other two K^+^ accumulation QTL (*QK.asl*-*2B.1* and *QK.asl*-*5A*), the beneficial allele was derived from Excalibur. In case of *QK.asl*-*2B.1*, the allele of interest enabled lines to accumulate 0.9 to 4.6% more K^+^ at four trial sites (Table 3). The 5A K^+^ accumulation QTL (*QK.asl*-*5A*) had increased K^+^ accumulation with the allele of interest at the Whitwarta low salt site in 2015 (Table 3).

### Effect of alleles on grain yield

The effect of the alleles of interest on grain yield for all the QTL is summarized in Table 3. For the shoot ion-independent QTL (*QG*_*(1***–***5)*_*.asl*-*7A*), the allele for improved growth under salinity treatment is derived from Excalibur and showed yield advantages only at the low and high salt sites at Whitwarta in 2014. However, a number of other QTL had a greater effect on yield at several low and high salt sites (Table 3). For the Na^+^ accumulation QTL on chromosome 7A (*QNa.asl*-*7A*), the lines carrying the Excalibur allele increased yield by 12% at the Whitwarta low salt site in 2015 and by 4 and 6.9% at the low and high salt sites, respectively, at Whitwarta in 2014 (Table 3). Of the four K^+^ accumulation QTL (*QK.asl*-*2B.1*, *QK.asl*-*2B.2*, *QK.asl*-*5A* and *QK:Na.asl*-*6A*), *QK.asl*-*2B.2* affected yield at all trial sites, and the other three QTL affected yield advantages at only four sites (Table 3).

### Screening of cultivars for shoot ion-independent tolerance

A selection of Australian bread wheat cultivars, as well as one landrace (BH1146), was screened to indicate the prevalence of a marker allele that was associated with the Excalibur like shoot ion-independent tolerance. Genotyping results of 44 accessions showed that the marker allele associated with shoot ion independence was present in five cultivars and the landrace (Supplementary Table 8). Of these accessions, two cultivars (Excalibur and Krichauff) had a mix of plants with (T:T) sensitive or (C:C) tolerant alleles.

### Predicted genes in QTL regions

All the marker sequences within the region of 2 LOD drop from the maximum likelihood value of the selected QTL (*QG*_*(1***–***5)*_*.asl*-*7A*, *QNa.asl*-*7A*, *QK.asl*-*2B.1*, *QK.asl*-*2B.2*, *QK.asl*-*5A*, *QK:Na.asl*-*6A*) were used for BLASTn searches to find scaffold sequences from the bread wheat NRGene genome assembly (International Wheat Genome Sequencing Consortium, https://wheat-urgi.versailles.inra.fr/Seq-Repository/Assemblies). As expected, SNP sequences had their best hits on the corresponding chromosomes, and thus, it was possible to retrieve matching scaffolds underlying the QTL.

The QTL for maintenance of shoot growth under salinity (*QG*_*(1***–***5)*_*.asl*-*7A*) were located on five wheat genome scaffolds (scaffold34994-1, scaffold98898, scaffold2159, scaffold75839 and scaffold56294) containing 207 expressed genes (Supplementary Table 9). Within this region, a sodium/hydrogen exchanger 7 (*NHX7*) also known as salt overly sensitive 1 (*SOS1*) and a potassium transporter 1 (KUP1) was shortlisted as potential candidate genes based on their role in Na^+^ and K^+^ homoeostasis, respectively (Olias et al. 2009; Sun et al. 2015; Ullah et al. 2016; Zhu 2002, 2003) (Table 4).

**Table 4:** List of potential candidate genes (Gene ID, Gene name) for each QTL, with the respective Munich Information Center for Protein Sequences (MIPS) annotation hit ID and rice annotation hit ID

QTL	Gene ID	Gene name	MIPS annotation hit ID	Rice annotation hit ID
*QG*_*(1***–***5)*_*.asl*-*7A*	Traes_7AL_8EB535289	Sodium/hydrogen exchanger 7	sp|Q9LKW9|NHX7_ARATH	LOC_Os12g44360.1
	Traes_7AL_65EAA4F5F	Potassium transporter 1	AT2G30070.1	LOC_Os06g45940.2
*QNa.asl*-*7A*	Traes_7AS_0EA301557	K^+^-insensitive pyrophosphate-energized proton pump	sp|Q8RCX1|HPPA_THETN	LOC_Os06g08080.1
	Traes_7AL_33B58D8DB	Sodium/hydrogen exchanger 7	sp|Q9LKW9|NHX7_ARATH	LOC_Os12g44360.1
	Traes_7AL_0E143E0C2	Cation transporter HKT1	sp|A2YGP9|HKT1_ORYSI	LOC_Os06g48810.1
	Traes_7AL_D35672777	Cation transporter HKT1	sp|A2YGP9|HKT1_ORYSI	LOC_Os06g48800.1
*QK.asl*-*2B.1*	Traes_2BS_FC456664B	Potassium transporter family protein	AT1G60160.1	LOC_Os07g32530.1
*QK.asl*-*2B.2*	Traes_2BL_E48A792E2	Potassium transporter family protein	AT1G60160.1	LOC_Os04g32920.4
	Traes_2BL_3198833F4	Cation transporter HKT1	sp|A2YGP9|HKT1_ORYSI	LOC_Os04g51830.1
	Traes_2BL_A34360A6C	Cation transporter HKT1	sp|A2YGP9|HKT1_ORYSI	LOC_Os04g51820.2
	Traes_2BL_4288159C3	Cation transporter HKT1	sp|A2YGP9|HKT1_ORYSI	LOC_Os04g51830.1
	Traes_2BL_7F4C72451	Cation transporter HKT1	sp|A2YGP9|HKT1_ORYSI	LOC_Os04g51830.1
	Traes_2BL_C71ACBCED	Potassium channel SKOR	sp|Q9M8S6|SKOR_ARATH	LOC_Os04g36740.1
	Traes_2BL_1356B2300	Chloride channel C	AT5G49890.1	ChrSy.fgenesh.mRNA.37
	Traes_2BL_8FFBE8AF6	Proline transporter 1	AT2G39890.1	LOC_Os03g44230.1
*QK.asl*-*5A*	Traes_5AL_51E31BF07	Two-pore potassium channel a	sp|Q850M0|KCO1_ORYSJ	LOC_Os03g54100.2
	Traes_5AL_ACFA5E386	Peptide transporter 3	AT5G46050.1	LOC_Os11g17970.1
*QK:Na.asl*-*6A*	Traes_6AL_77B5B62A7	H^+^-ATPase 6	AT2G07560.1	LOC_Os06g08310.1
	Traes_6AL_B830C4A81	Plasma membrane ATPase 4	sp|Q03194|PMA4_NICPL	LOC_Os02g55400.1

For the Na^+^ accumulation QTL (*QNa.asl*-*7A*), a total of 12 scaffolds (scaffold9784, scaffold78307, scaffold12617, scaffold18535, scaffold49337, scaffold36269-2, scaffold156548, scaffold107677, scaffold51305, scaffold143236, scaffold18984 and scaffold4982) with 986 expressed genes were retrieved (Supplementary Table 9). A pyrophosphate-energized proton pump (H^+^ pyrophosphatase), sodium/hydrogen exchanger 7 (*NHX7*) and *HKT* genes (*HKT2;1* and *HKT2;*4) were shortlisted as candidate genes based on their role in salt tolerance (Almeida et al. 2013; Gaxiola et al. 2001; Huang et al. 2008; Olias et al. 2009; Ullah et al. 2016; Zhu 2002, 2003) (Table 4).

For the K^+^ accumulation QTL (*QK.asl*-*2B.1*), a total of six scaffolds (scaffold32978, scaffold76358-1, scaffold88768, scaffold13132, scaffold18146 and scaffold60636) with 208 expressed genes were retrieved (Supplementary Table 9). A K^+^ transporter (*KUP12*) was shortlisted as a candidate gene based on its function (Quintero and Blatt 1997) (Table 4). For the second K^+^ accumulation QTL on chromosome 2B (*QK.asl*-*2B.2*), a total of 18 scaffolds with 873 expressed genes were retrieved (Supplementary Table 9) with many potential candidates including *HKT* genes (*HKT1;1* and *HKT1;*4), as well as genes encoding a number of K^+^ transporters (including stelar K^+^ outwardly rectifying channel, SKORs), Cl^−^ channels, protein kinases and proline transporters (Table 4).

For K^+^ accumulation QTL (*QK.asl*-*5A*), a total of two scaffolds (scaffold45906 and scaffold21633) were identified having 123 expressed genes (Supplementary Table 9) with two obvious candidate genes being a two-pore potassium (TPK) channel and a peptide transporter (Table 4). The TPK channel is known to be involved in K^+^ homoeostasis (Isayenkov et al. 2011), while the peptide transporter 3, also known as *AtPTR3*, was shown to be involved in seed germination under salt stress (Karim et al. 2005).

For the K^+^:Na^+^ QTL (*QK:Na.asl*-*6A*), a total of three scaffolds (scaffold32586, scaffold12078 and scaffold82975) with 188 expressed genes were retrieved. Within this region, there are plasma membrane proton pump ATPase (H^+^-ATPase 4 and H^+^-ATPase 6) which are selected as candidate genes based on their role in salt tolerance (Gévaudant et al. 2007; Morsomme and Boutry 2000) (Supplementary Table 9).

## Discussion

In this study, non-destructive imaging technology was used to map QTL affecting salt tolerance sub-traits in a glasshouse experiment for a DH mapping population of hexaploid wheat. We detected 10 unique QTL under salt and control conditions. These were distributed across five chromosomes: 1A, 2B, 5A, 6A and 7A (Table 2). Some of these QTL were confirmed to have effects in field experiments. At six of nine field trial sites, which varied in their level of salinity stress, alleles for Na^+^ exclusion and/or K^+^ maintenance at three QTL (*QNa.asl*-*7A*, *QK.asl*-*2B.2* and *QK:Na.asl*-*6A*) were able to exclude more Na^+^ or accumulate more K^+^ (Table 3). At all field sites, the QTL *QK.asl*-*2B.2* had the largest improvement on grain yield. Other QTL also showed yield improvements but only at selected field sites. Some other QTL for Na^+^ exclusion and K^+^ maintenance that were detected in the glasshouse were replicated in the field but did not affect grain yield (Table 3).

Four QTL (*QK.asl*-*2B.1, QK.asl*-*2B.2, QK.asl*-*5A* and *QK:Na.asl*-*6A*) were associated with K^+^ accumulation under salinity treatment in the glasshouse. *QK.asl*-*2B.2*, the second most significant QTL detected for leaf K^+^ accumulation in the glasshouse, was validated in both low and high salinity field sites (Table 3). This QTL was consistently associated with grain yield effects of 1–13%, averaging 4% (Table 3). Maintenance of high levels of K^+^ in a plant cell under salinity stress, which has previously been reported to be an important sub-trait of salt tolerance (Cuin et al. 2008, 2009; Shabala 2013), vital metabolic processes such as protein synthesis (Blaha et al. 2000; Wyn Jones et al. 1979), key enzyme activity (Bhandal and Malik 1988) and signalling mechanisms (Shabala 2017).

Among 873 genes within the interval of *QK.asl*-*2B.2*, there are two predicted *HKT* genes (*HKT1;1* and *HKT1;*4) (Huang et al. 2008), several predicted genes that encode K^+^ transporters (including stelar K^+^ outwardly rectifying channel, SKORs), Cl^−^ channels, protein kinases and proline transporters (Table 4). *TmHKT1;4* (*Nax1*) have been shown to improve Na^+^ exclusion (and K^+^ retention) when introgressed into durum wheat (James et al. 2006, 2012) but did not improve yield in the field (James et al. 2012). When *TmHKT1;4* was introgressed into bread wheat, a similar ionic phenotype was observed (James et al. 2011). Enhanced control over Na^+^ and K^+^ transport in bread wheat would allow for better maintenance of high levels of K^+^ in a plant under salinity stress and may be associated with the *QK.asl*-*2B.2* detected in this study.

Two other QTL that were detected for K^+^ accumulation (*QK.asl*-*2B.1*; *QK.asl*-*5A*) in glasshouse experiments were not validated at field sites. Each of these was detected in a region with a known developmental gene: the photoperiod-insensitive gene *Ppd*-*B1 (QK.asl*-*2B.1)*, or the vernalization gene *Vrn*-*A1* (*QK.asl*-*5A*). In other studies, QTL for multiple traits including tiller number, seedling biomass, chlorophyll content, plant height and yield have been detected in the same genetic region as *Vrn*-*A1*, in wheat grown in saline and/or non-saline treatments (De León et al. 2011; Genc et al. 2010; Hill et al. 2015; Kato et al. 2000; Kumar et al. 2007).

Although *QK.asl*-*5A* seems to coincide with *Vrn*-*A1*, it was detected in salt-stressed treatments but not in the control treatment (Table 2). This indicates that genes other than *Vrn*-*A1* may be responsible for the *QKasl*-*5A* effect. These may include genes that are orthologous to Arabidopsis genes that encode an element of the *two*-*pore potassium* (*TPK*) channel (Hartley and Maathuis 2016; Isayenkov et al. 2011) or the *peptide transporter 3* (*AtPTR3*) (Table 4). When expressed ubiquitously, the Arabidopsis *TPK1* affects salt tolerance (Latz et al. 2013), stomatal closure (Gobert et al. 2007; Ward and Schroeder 1994) and general K^+^ homoeostasis (Gobert et al. 2007; Hamamoto et al. 2008). The other gene, an ortholog of *AtPTR3*, belongs to the *nitrate excretion transporter* (*NAXT*) sub-family of the *nitrate transporter 1/peptide transporter* (*NRT1/PTR*) family (*NPF*) (Léran et al. 2014; Li et al. 2017; Segonzac et al. 2007; Tsay et al. 2007). In Arabidopsis, around 50 putative NRT1/PTR-type transporters have been identified (Karim et al. 2005), some of which have been implicated in salt tolerance mechanisms (Karim et al. 2005; Li et al. 2017). To determine whether the K^+^ QTL mapped in this region is due to *Vrn*-*A1* itself or some other gene(s), more lines with recombination events in this region would need to be phenotyped and genotyped. Previously, this approach was used to separate the effect of the *Nax3* (*HVP10*) gene from the flowering time gene *HvFT* in a barley mapping population (Shavrukov et al. 2013).

A QTL on chromosome 7A for leaf Na^+^ exclusion (*QNa.asl*-*7A*) (Table 2) was detected under 100 mM salt stress in the glasshouse. This leaf Na^+^ exclusion QTL was verified in most field sites, with lines carrying the same marker alleles as Excalibur having up to 34% lower leaf Na^+^ than those with Kukri alleles (Table 3). This QTL is in the same region as a leaf Na^+^ exclusion QTL previously detected on chromosome 7A in a Cranbrook × Halberd population and Excalibur × Kukri DH (Edwards 2012; Shavrukov et al. 2011). Within this QTL interval, several salt tolerance genes such as a proton pumping pyrophosphate (H^+^-pyrophosphatase), sodium/hydrogen exchanger 7 (*NHX7*) and *HKT* genes (*HKT2;1 and HKT2;4*) have been found (Table 4). The H^+^-pyrophosphatase is the closest of these genes to the peak of the QTL. Enhanced expression of H^+^-pyrophosphatase (Gaxiola et al. 2001; Park et al. 2005; Pasapula et al. 2011; Schilling et al. 2014), particularly in conjunction with enhanced sodium/hydrogen exchanger gene expression (Bhaskaran and Savithramma 2011; Gaxiola et al. 1999; Zhao et al. 2006), such as *NHX7* which is also within this QTL, has been shown to enhance salinity tolerance. The *HKT* genes are the most distant of these four genes from the peak; however, previous studies (Ariyarathna et al. 2014, 2016; Huang et al. 2008) have also reported the presence of these genes in the same region as QTL for leaf Na^+^ concentration and yield measurements under different saline environments (Genc et al. 2010, 2013). Identification of leaf Na^+^ exclusion QTL in current and previous studies (Edwards 2012; Genc et al. 2010, 2013; Shavrukov et al. 2011) on chromosome 7A and the presence of *HKT* genes (*HKT2;1 and HKT2;4*) in this region suggest the importance of this locus in salt tolerance. TaHKT2;1 has been described as a Na^+^-coupled K^+^ transporter protein (Gassmann and Schroeder 1994; Sato et al. 2014; Schachtman and Schroeder 1994) which is involved in balancing K^+^:Na^+^ ratio in the plant. Care is needed in interpreting these results as the DAWN and POTAGE bioinformatics tools that were used here to identify candidate genes, only search databases that contain wheat genes expressed under control treatments. Any genes involved in salinity tolerance that are expressed only at low levels in non-salt-stressed plant would not be represented in these databases. With transcript analysis of salt-stressed wheat, it might be possible to identify additional candidate genes within this region which have not been captured by the current bioinformatics analysis. Additionally, fine mapping of the region will help reduce the number of genes.

Very few studies have investigated the impact of Na^+^ exclusion alleles on yield in the field. Despite the *QNa.asl*-*7A* allele being associated with leaf Na^+^ exclusion, this allele was not correlated with yield improvement in low-to-moderate saline sites (Table 3). The lack of a positive correlation between leaf Na^+^ exclusion and yield in bread wheat has been noted in other studies (Genc et al. 2013). It is apparent that improving salinity tolerance of bread wheat across a range of salinity environments is not simply a matter of enhancing leaf Na^+^ exclusion in bread wheat.

Na^+^ exclusion has been found to improve salinity tolerance and yield performance under increasing salinity for the high shoot Na^+^ accumulating crops durum wheat (James et al. 2012; Munns et al. 2012) and barley (Tilbrook et al. 2017). A derivative of the durum cultivar Tamaroi containing the *Nax2* locus (*TmHKT1;5*-*A*) had 25% higher yield in a highly saline area (EC_e_ = 14.8 dS/m) compared to Tamaroi without the locus (Munns et al. 2012). While leaf Na^+^ exclusion did not improve the yield of bread wheat in our study, the previous success with Na^+^ exclusion in durum wheat in more saline environments indicates that it can improve yield in certain crops/cultivars under specific environments. It is conceivable that, as bread wheat is already a good shoot Na^+^ excluder, enhancing this capability in bread wheat is not necessarily going to result in large improvements in salinity tolerance. Shoot Na^+^ exclusion may therefore be a more important mechanism to improve the yield stability of crop species that accumulate high concentrations of shoot Na^+^.

A novel QTL for shoot ion-independent tolerance was detected on chromosome 7A (*QG*_*(1***–***5)*_*.asl*-*7A*). To date there is no published QTL about the presence of shoot tolerance mechanisms not related to shoot ion concentration in wheat; however, QTL for growth traits have recently been detected in rice on chromosomes 1, 3, 5, 8 and 11 (Al-Tamimi et al. 2016; Campbell et al. 2015). A QTL for osmotic regulation has previously been mapped on chromosome 7A (Morgan and Tan 1996), but not in the same region as the shoot ion-independent tolerance QTL reported here. Analysis of the shoot ion-independent QTL by POTAGE and DAWN identified 207 genes within the region. One of these genes encodes a K^+^ transporter (*KUP1*) which is important in maintaining potassium homoeostasis (Hu et al. 2005; Maathuis and Amtmann 1999; Sun et al. 2015), while the second one (*NHX7*) is involved in Na^+^ transport (Olias et al. 2009; Ullah et al. 2016; Zhu 2002, 2003). Further fine mapping is required to reduce the number of candidate genes linked to the phenotype.

*QG*_*(1***–***5)*_*.asl*-*7A* had no large effect on yield in the field, despite having a significant effect in the glasshouse (LOD = 5, phenotypic variation (*R*^2^) = 14.1%). Only at Whitwarta (low and high salt) in 2014 was there any yield improvement (Table 3). The lack of yield improvement at multiple sites may be due to the complex nature of the shoot ion-independent tolerance mechanism and the influence of other multiple environmental and biotic factors. In addition to salinity, plants in a saline field are also affected by other abiotic stresses, with the most common of these being drought and waterlogging (Colmer et al. 2005; Genc et al. 2013; Mittler 2006; Munns 2002; Munns et al. 2016; Tester and Davenport 2003). Concurrent occurrence of two or more stresses is considered more detrimental to plant performance than an individual stress alone (Barrett-Lennard 2003; Mittler 2006; Pandey et al. 2015). Some of our trial sites were exposed to harsher environmental conditions at the start of the growing season (such as waterlogging and low water availability), while others experienced low rainfall towards the end of the growing season (Supplementary Table 2 and Supplementary Figure 1).

A direct measurement of shoot ion-independent tolerance in the field is lacking to confirm the phenotype observed in the glasshouse. Alleles for early growth response to salinity stress may improve biomass production during seedling establishment. Technology to measure plant phenotypes, such as biomass, water use, shoot health (senescence) and canopy temperature, late in the growing season is now possible using platforms, such as drones, phenotowers and phenomobiles fitted with a selection of RGB, LiDAR and multispectral cameras (Araus and Cairns 2014; Deery et al. 2014, 2016; Furbank and Tester 2011; Großkinsky et al. 2015; Losos et al. 2013; Rascher et al. 2011). However, robust non-destructive phenotyping techniques to accurately measure early plant growth over time in the field are still in their infancy. As new technologies become available, it will be possible to measure shoot ion-independent phenotypes in young plants in the field to determine whether glasshouse shoot ion-independent tolerance can be translated in the field.

We cannot rule out shoot ion-independent tolerance as an important trait. When a marker for *QG*_*(1***–***5)*_*.asl*-*7A* was assayed on 44 varieties of bread wheat and one landrace, the tolerance-associated allele present in Excalibur-198 was detected in Axe, BH1146 (a landrace), Estoc and Halberd (Supplementary Table 8). The allele was also present in the heterogeneous Excalibur and Krichauff cultivars; plants of either of these cultivars could have the Excalibur-198 or the Kukri allele. Of these six accessions, four (Krichauff, Excalibur, Halberd and BH1146) have been reported as having salinity tolerance (Genc et al. 2007; Khokhar et al. 2017; Takahashi et al. 2015; Zhu et al. 2016). Future work should focus on identification of haplotypes and their link to salinity tolerance in this QTL locus. It is possible that shoot ion-independent tolerance will have an improvement on crop growth when combined with other salinity tolerance sub-traits, such as leaf Na^+^ exclusion and K^+^ maintenance (Rajendran et al. 2009; Tilbrook et al. 2017). The development of crops with improved yield in hostile environments will require the pyramiding of multiple traits including, but not exclusive to, tolerance to boron, drought, heat, frost, waterlogging, pH, nematodes and multiple diseases and pathogens, as well as optimizing phenology while maintaining grain yield and quality.

In summary, a number of QTL for salinity tolerance mechanisms identified in the glasshouse were validated in the field. Promisingly, several of these loci were also linked to improved yield in up to nine field sites, across multiple years. QTL for shoot ion-independent tolerance were also detected for the first time in glasshouse grown bread wheat. Future work should focus on developing robust measurements for measuring shoot ion-independent tolerance in the field and positional cloning of the gene(s) responsible for all the identified QTL.

### Author contribution statement

MA. performed genotyping, QTL analysis and experiments, R.S. and S.R conducted field trials and performed data analysis, C.B, K.D. and H.R. completed experimental design and statistical analysis, R.S. J.T, E.B-L, L.S, C.T and S.R. performed glasshouse and field experiments. A.G contributed to QTL analysis, D.M assisted in map construction, D.F and A.P contributed to genetic analysis and supervision and B.B, M.G, M.T and S.R conceived the study. All authors contributed to writing and reviewing the manuscript.

## Electronic supplementary material

Below is the link to the electronic supplementary material.

Supplementary material 1 (DOCX 1430 kb)

Supplementary material 2 (XLSX 9 kb)

Supplementary material 3 (XLSX 14 kb)

Supplementary material 4 (XLSX 1447 kb)

Supplementary material 5 (XLSX 25 kb)

Supplementary material 6 (XLSX 309 kb)
