# Supplementary material for: Mapping of novel salt tolerance QTL in an Excalibur × Kukri doubled haploid wheat population
Source: Theor Appl Genet. 2018 Jul 30;131(10):2179–96. doi: 10.1007/s00122-018-3146-y (PMC6154029; doi:10.1007/s00122-018-3146-y)
Supplement: Supplementary file 1 — Supplementary material 1 (DOCX 1430 kb) [file 122_2018_3146_MOESM1_ESM.docx]

**Supplementary Data**

**Supplementary Table 2.** Field experiment locations, total growing season rainfall (April to November) and mean minimum (min) and maximum (max) temperature (ºC) in the growing season (April to November) for all trials. Rainfall data for Whitwarta, Cunderdin, and Coomandook were from taken from weather station 21112, 10286, and 25503, respectively whereas the temperature data for the three trial sites were taken from weather station 22015, 10286, and 24518 (<http://www.bom.gov.au/climate/>).

| **Year** | **Location** | **Latitude** | **Longitude** | **Rainfall April-Nov (mm)** | **Max Temperature April-Nov (°C)** | **Min Temperature April-Nov (°C)** |
| --- | --- | --- | --- | --- | --- | --- |
| 2014 | Whitwarta  Cunderdin | 34.1000° S  31.6363° S | 138.3333° E  117.2419° E | 284  351 | 21  23 | 10  8 |
| 2015 | Whitwarta  Coomandook  Cunderdin | 34.1000° S  35.4414° S  31.6363° S | 138.3333° E  139.7732° E  117.2419° E | 271  245  209 | 20  19  24 | 9  9  8 |

**Supplementary Table 3**

Lines of best fit and R^2^ values for the measured EM38 value (mS/m) at 0 to 50 cm in August at Cunderdin 2014 and September at Cunderdin 2015; and 0 to 100 cm in October at Whitwarta in 2014 and 2015 and Coomandook 2015 versus the measured soil electrical conductivity (EC_1:5_ dS/m) within the low and high salinity sites at two soil depths (0-25 cm and 25-50 cm). Lines of best fit and the measured EM38 values for each plot were used to derive an EC_1:5_ value for each plot which was used as a covariate in the statistical analysis of field data. nd = no data.

| **Year** | **Location** | **Site** | **Soil Depth** | **Line of Best Fit** | **R^2^** | **P-value** |
| --- | --- | --- | --- | --- | --- | --- |
| 2014 | Whitwarta | Low Salt | 0-25 cm | y = 0.0007x + 0.0733 | 0.2523 | <0.05 |
|  |  |  | 25-50 cm | y = 0.0037x – 0.1128 | 0.5873 | 0.192 |
|  |  | High Salt | 0-25 cm | y = 0.0019x – 0.1113 | 0.3714 | 0.375 |
|  |  |  | 25-50 cm | y = 0.0073x – 0.8130 | 0.5757 | <0.05 |
|  | Cunderdin | Low Salt | 0-25 cm | nd | nd | nd |
|  |  |  | 25-50 cm | nd | nd | nd |
|  |  | High Salt | 0-25 cm | y = 0.0099x - 1.4209 | 0.8647 | <0.001 |
|  |  |  | 25-50 cm | y = 0.0115x - 1.1012 | 0.7880 | <0.001 |
| 2015 | Whitwarta | Low Salt | 0-25 cm | y = 0.00047x + 0.0623 | 0.2938 | <0.001 |
|  |  |  | 25-50 cm | y = -0.1162x + 120.14 | 0.0036 | 0.146 |
|  |  | High Salt | 0-25 cm | y = 0.0073x – 0.9994 | 0.6859 | <0.001 |
|  |  |  | 25-50 cm | y = 0.0084x – 1.0714 | 0.6794 | <0.001 |
|  | Cunderdin | Low Salt | 0-25 cm | y = 0.0021x - 0.0409 | 0.8233 | <0.001 |
|  |  |  | 25-50 cm | y = 0.0065x - 0.2051 | 0.9094 | <0.001 |
|  |  | High Salt | 0-25 cm | y = 0.0075x - 1.1201 | 0.7325 | <0.001 |
|  |  |  | 25-50 cm | y = 0.0084x - 0.7925 | 0.7139 | <0.001 |
|  | Coomandook | Low Salt | 0-25 cm | y = 0.0005x – 0.0426 | 0.2324 | 0.266 |
|  |  |  | 25-50 cm | y = 0.0015x – 0.1433 | 0.2014 | 0.211 |
|  |  | High Salt | 0-25 cm | 9.8344x - 1038.3 | 0.4684 | <0.05 |
|  |  |  | 25-50 cm | 13.25x - 1428.3 | 0.4350 | <0.05 |

| Linkage groups | Total markers | Length (cM) | Marker density (cM/markers) |
| --- | --- | --- | --- |
| 1A | 246 | 141.34 | 0.57 |
| 1B | 228 | 120.82 | 0.53 |
| 1D | 69 | 135.27 | 1.96 |
| 2A | 199 | 164.96 | 0.83 |
| 2B | 369 | 157.82 | 0.43 |
| 2D | 71 | 77.62 | 1.09 |
| 2DS | 20 | 15.53 | 0.78 |
| 3A | 199 | 166.64 | 0.84 |
| 3B | 223 | 206.38 | 0.93 |
| 3DL | 21 | 1.94 | 0.09 |
| 3DS/L | 18 | 84.65 | 4.70 |
| 4A | 240 | 169.87 | 0.71 |
| 4B | 0 | 0.00 | 0.00 |
| 4D | 13 | 40.35 | 3.10 |
| 5A | 197 | 215.71 | 1.09 |
| 5B | 295 | 172.69 | 0.59 |
| 5DL | 25 | 18.95 | 0.76 |
| 5DS/L | 19 | 96.25 | 5.07 |
| 6A | 199 | 100.98 | 0.51 |
| 6B | 224 | 170.46 | 0.76 |
| 6DLB | 36 | 17.24 | 0.48 |
| 6DLT | 8 | 7.13 | 0.89 |
| 6DS | 6 | 4.78 | 0.80 |
| 7A | 296 | 180.30 | 0.61 |
| 7B | 246 | 132.41 | 0.54 |
| 7DL | 7 | 24.01 | 3.43 |
| 7DS | 11 | 53.38 | 4.85 |
| 7DS/L | 18 | 29.08 | 1.62 |
| A genome | 1576 | 1139.80 | 0.72 |
| B genome | 1585 | 960.57 | 0.61 |
| D genome | 342 | 606.19 | 1.77 |
| Total | 3503 | 2706.56 | 0.77 |

**Supplementary Table 5.** Linkage groups, marker distribution, density and length of the Excalibur × Kukri DH genetic linkage map. The short and long arms of the chromosomes are labelled S and L, respectively. No polymorphisms were detected on chromosome 4B in this population.

**Supplementary Table 8.** Genotypes of 44 bread wheat cultivars and a landrace determined by assaying the marker X2279012_58AC on 3 plants of each cultivar. Varieties in this seed stock that contain genotypes of both T:T (sensitive) and C:C (tolerant) are highlighted with an *.

| **Marker X2279012_58AC** | **Cultivar** |
| --- | --- |
| C:C (Tolerant) | Axe, BH1146 (landrace), Estoc, Excalibur*, Halberd, Krichauff* |
| T:T (Sensitive) | Batavia, Baxter, Bremer, Calingiri, Carnamah, Cobra, Condo, Corack, Emu Rock, Envoy, Excalibur*, Gladius, Gregory, Hartog, Hydra, Impala, Janz, Kukri, Krichauff*, Lancer, Livingston, LPB-Flanker, LPB-Arrow, Mace, Magenta, Pelsart, Scout, Spitfire, Sunco, Sunmate, Suntop, Tammarin Rock, Trojan, Ventura, Viking, Wallup, Westonia, Wyalkatchem, Yitpi, Zen |


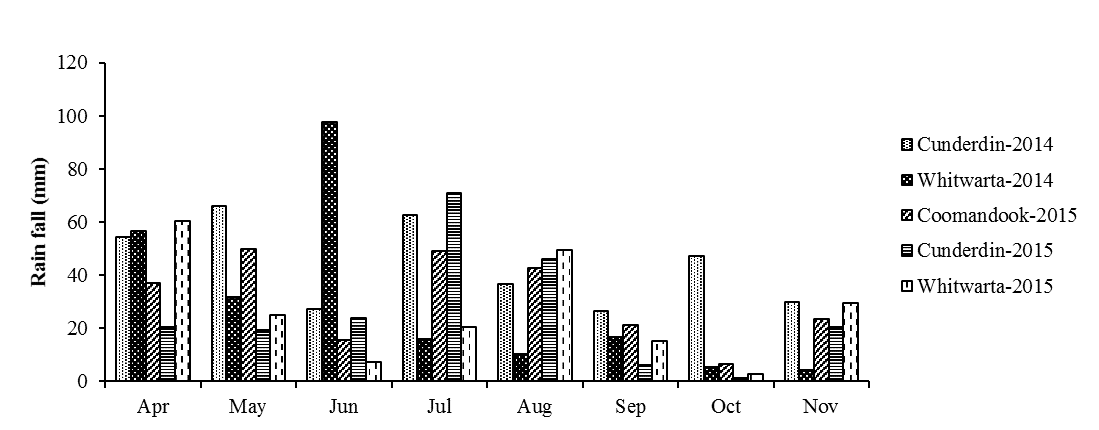


(a)


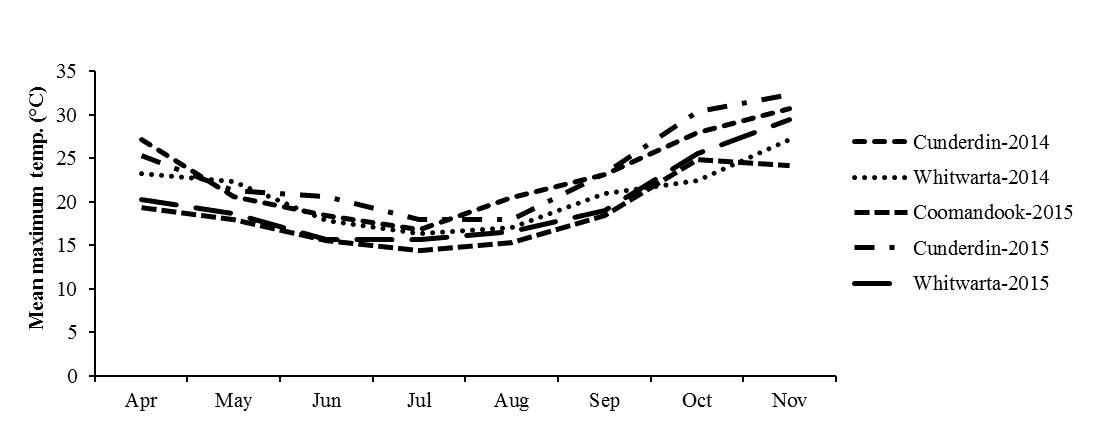


(b)

**Supplementary Figure 1.** (a) Average total rainfall (mm) for each month and (b) maximum daily temperature (°C) for each month (b) at trial sites for the year 2014 and 2015. Rainfall data for Cunderdin, Whitwarta and Coomandook were from weather station 10286, 21112 and 25503, respectively whereas the temperature data for the three trial sites were taken from nearest weather station 10286, 22015 and 24518 (<http://www.bom.gov.au/climate/>).

**Whitwarta**

**2014**

**Soil Depth: 0-50 cm**

**July**

**October**

**Soil Depth: 0-100 cm**

**October**

**July**

**Low Salt**

**High Salt**


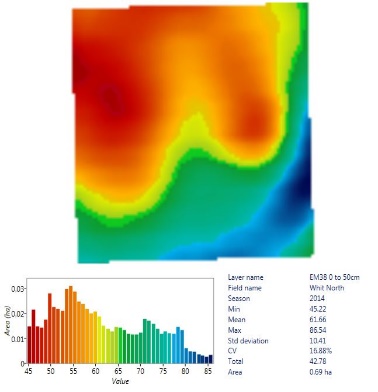


**45 to 87 mS/m**


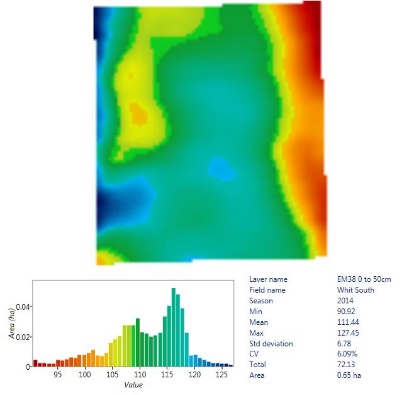


**91 to 127 mS/m**


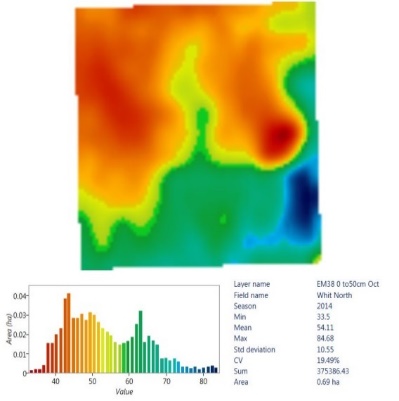

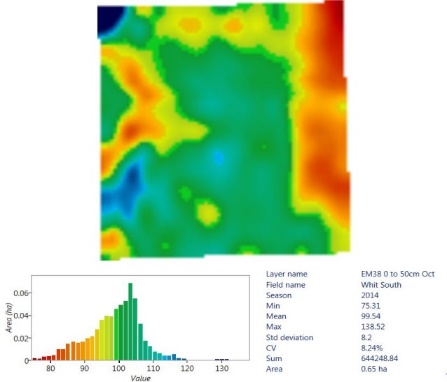


**75 to 139 mS/m**

**34 to 54 mS/m**


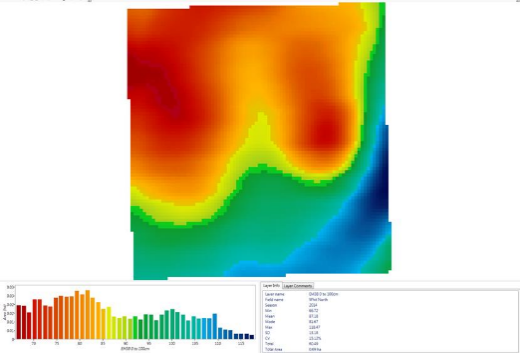

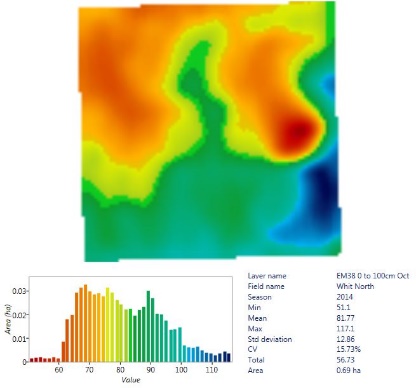

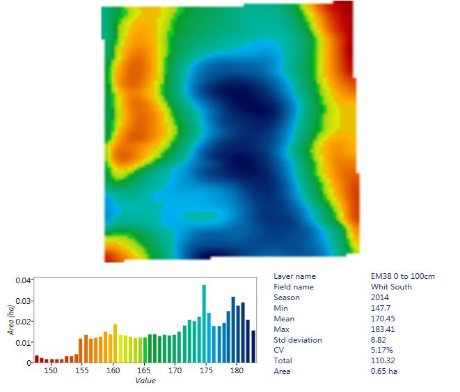

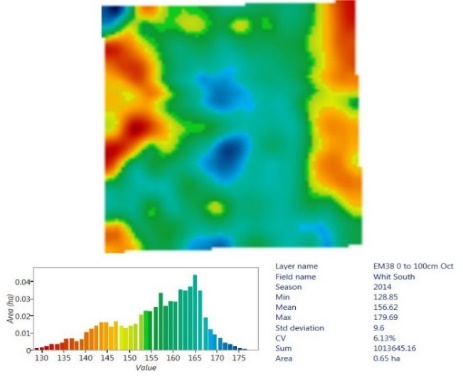


**67 to 119 mS/m**

**51 to 117 mS/m**

**148 to 183 mS/m**

**129 to 180 mS/m**

(a)


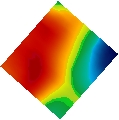

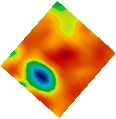

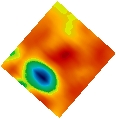

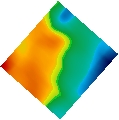

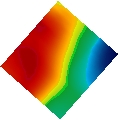

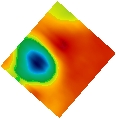


**63 to 207 mS/m**

**23 to 88 mS/m**

**42 to 95 mS/m**

**104 to 301 mS/m**

**134 to 263 mS/m**

**31 to 63 mS/m**

**Whitwarta**

**2015**

**Low Salt**

**High Salt**


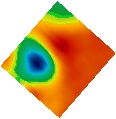


**89 to 199 mS/m**


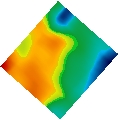


**17 to 59 mS/m**

(b)


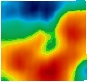

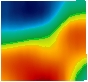

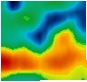

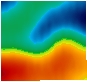

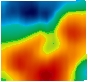

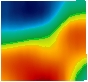

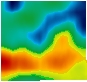

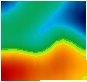


**76 to 192 mS/m**

**71 to 117 mS/m**

**74 to 104 mS/m**

**79 to 176 mS/m**

**110 to 165 mS/m**

**113 to 156 mS/m**

**114 to 215 mS/m**

**118 to 208 mS/m**

**Coomandook**

**2015**

**Low Salt**

**High Salt**

(c)

(d)

**Cunderdin**

**2014**

**Soil Depth: 0-100 cm**

**Soil Depth: 0-50 cm**

**August(2014), September(2015)**

**August(2014), September(2015)**

**June**

**June**

**High Salt**

mS/m

(e)

**Cunderdin**

**2015**

**Low Salt**

nd

nd

**High Salt**

mS/m

**Supplementary Figure 2.** EM38 maps showing the apparent electrical conductivity (EC_a_; mS/m) of low and high salt sites at two soil depths (0 to 50 cm and 0 to 100 cm) and across two months June and August (Cunderdin 2014), June and September (Cunderdin 2015), July and October (Whitwarta 2014 and 2015 and Coomandook in 2015). (a) Whitwarta 2014, low salt = 0.69 ha and high salt = 0.65 ha (b) Whitwarta 2015, low salt = 0.70 ha and high salt = 0.68 ha (c) Coomandook 2015, low salt = 0.68 ha and high salt = 0.68 ha (d) Cunderdin 2014 and (e) Cunderdin 2015. Red = low EC_a_ and blue = high EC_a_ within Whitwarta and Coomandook sites but follow a different scale between each site (low and high salt) – refer to the EC_a_ values provided under each figure for interpretation of EM38 maps. EC_a_ has been grouped from 0-100 (blue), 100-200 (red), 200-300 (green) and 300-400 (purple) mS/m in the low and high salt sites at Cunderdin with values comparable between sites (low and high salt). No EM38 data was recorded for the low salt site at Cunderdin in 2014 at both time-points or at the low salt Cunderdin site in June 2015. nd = no data.
